# Recurrent Shoulder Posteroinferior Subluxation Status Post Reverse Remplissage

**DOI:** 10.7759/cureus.7522

**Published:** 2020-04-03

**Authors:** Adam J Engel, William A Forshee, Christopher Wasyliw, Kurt Scherer

**Affiliations:** 1 Diagnostic Radiology, Florida Hospital-Orlando, Orlando, USA; 2 Radiology, Brandon Regional Hospital, Brandon, USA

**Keywords:** reverse remplissage, mclaughlin, reverse hill-sachs deformity, seizure, posterior dislocation, glenohumeral, instability, shoulder, humeral head

## Abstract

Posterior shoulder dislocation is an uncommon injury that typically follows intense contraction of the external rotator muscles, such as from seizure activity, high-velocity trauma, or intense electrical shock. The diagnosis is often missed or delayed, leading to complications such as functional deficits or osteonecrosis of the humeral head. Closed reduction can be utilized following an initial occurrence, however, repeated insult to the glenohumeral joint may lead to posterior instability. A reverse Hill-Sachs lesion, a vertical impacted fracture of the anteromedial aspect of the humeral head, can occur. Surgical treatment options for posterior instability include the modified McLaughlin procedure also known as the reverse remplissage procedure. Unfortunately, the success rates of this procedure are controversial.

## Introduction

Posterior shoulder dislocation is an injury generally following an intense contraction of the external rotator muscles, which can be secondary to direct trauma, seizure activity, or following intense electrical shock [1]. Posterior shoulder dislocation is a less common cause of shoulder instability and only accountable for approximately 2% to 10% of shoulder dislocations [2]. Although awareness is increasing, this diagnosis is often overlooked, being missed in up to 50% of cases, leading to complications such as functional incapacity or osteonecrosis of the humeral. Repeated insult to the glenohumeral joint can lead to chronic posterior instability [1,3-4].

## Case presentation

**Figure 1:**
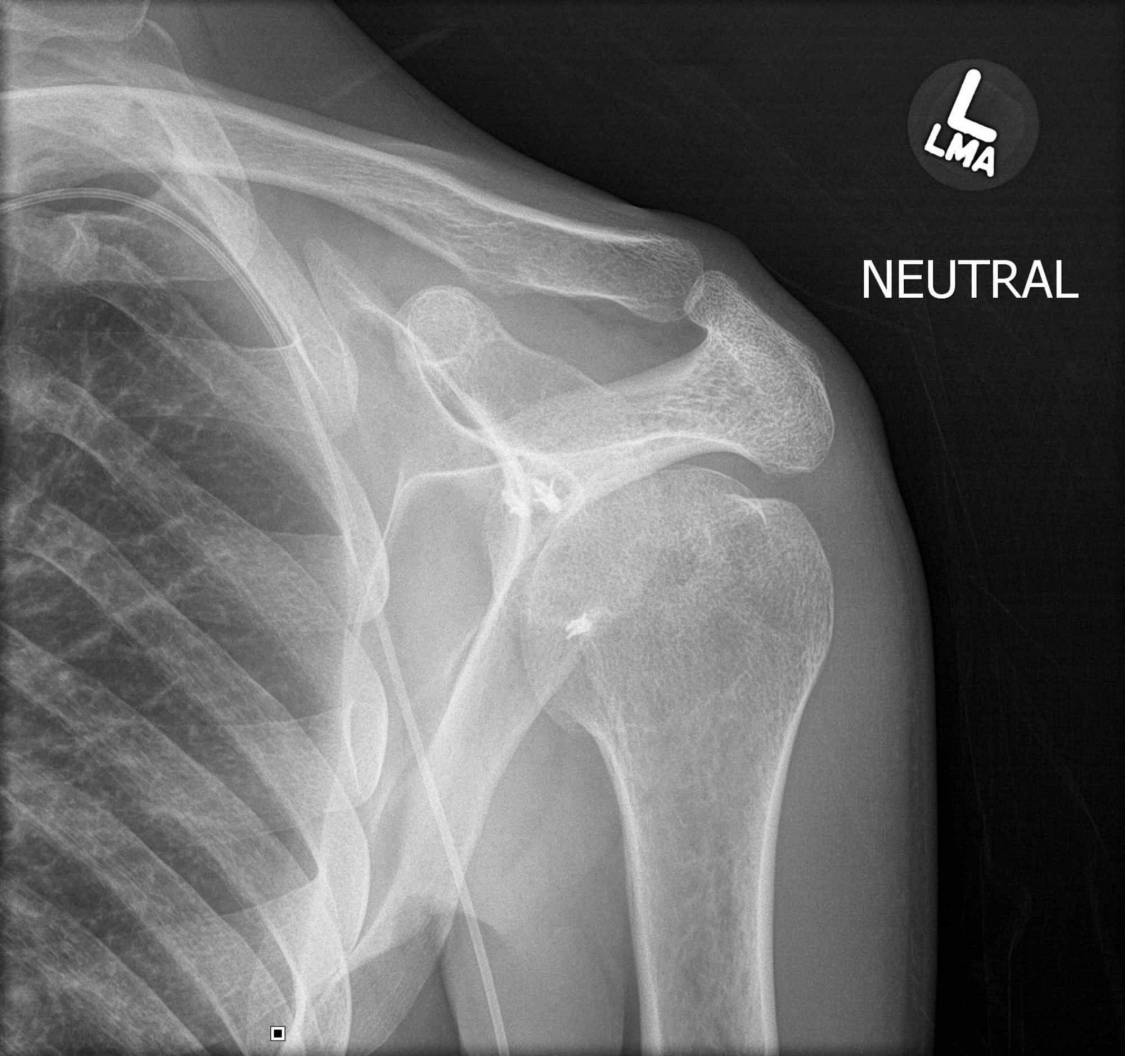
AP internal rotation radiograph of the shoulder showing posteroinferior subluxation of the humeral head AP: anteroposterior

**Figure 2:**
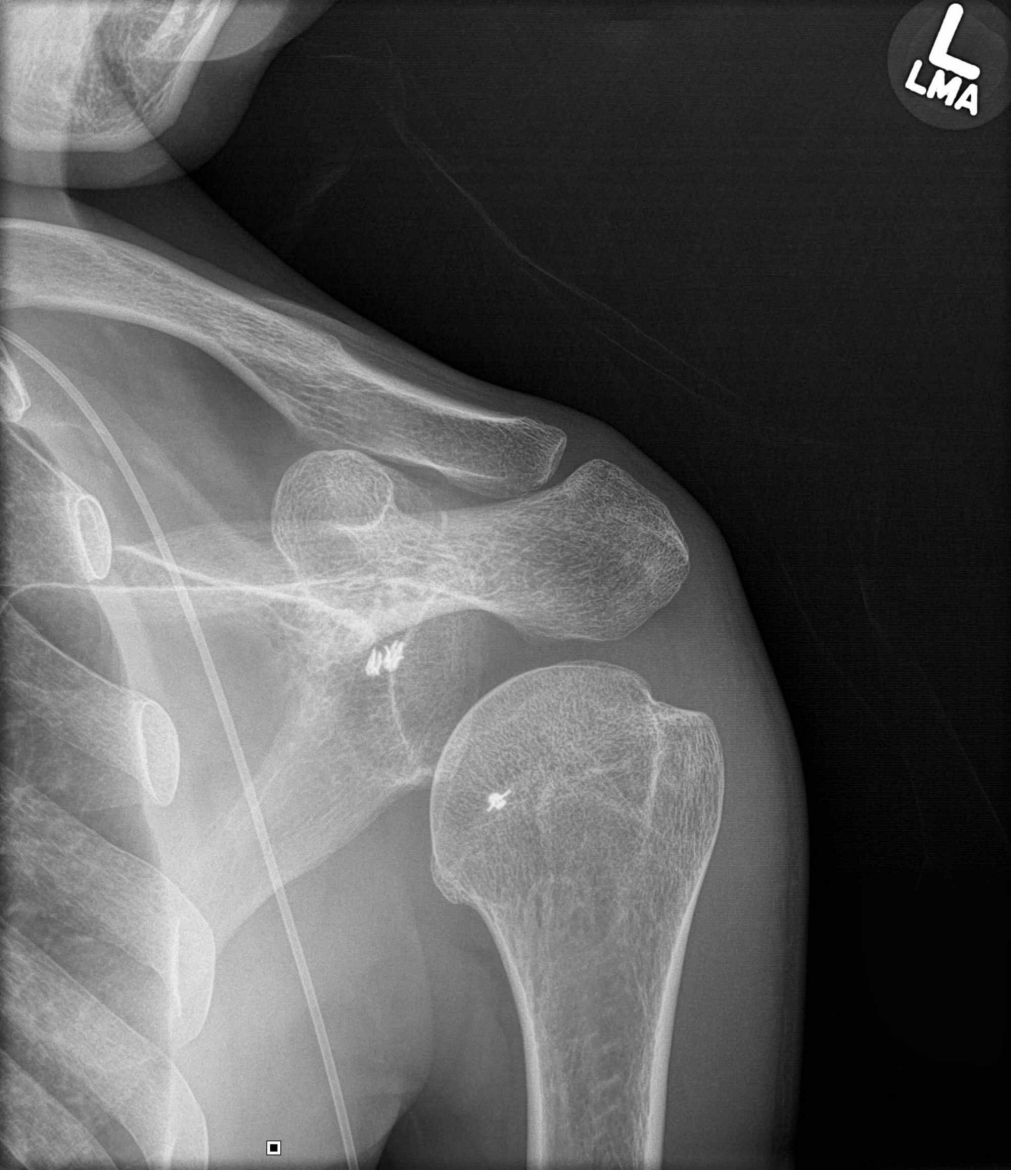
Grashey view radiograph of the shoulder showing posteroinferior subluxation of the humeral head.

We present a 39-year-old male with a history of chronic seizures with a new seizure on postoperative Day 2 following an open abdominal procedure (Figures 1-2). The patient underwent a McLaughlin type remplissage procedure for posterior instability 13 years prior to this presentation. Unfortunately, success rates for this procedure are variable, and our patient continued to experience seizure-related posterior dislocations following the procedure. An anteroposterior (AP) internal rotation shoulder radiograph (Figure 1) shows inferior subluxation of the humeral head with a less well-visualized degree of posterior subluxation.

In posterior dislocation, the contour of the humeral head resembles a lightbulb when viewed from the front, which is aptly described as the “lightbulb sign” [5]. On the Grashey view (Figure 2), or “true” AP view, one can inspect the integrity of the glenohumeral joint best by preventing overlap of the humeral head over the glenoid [6].

**Figure 3:**
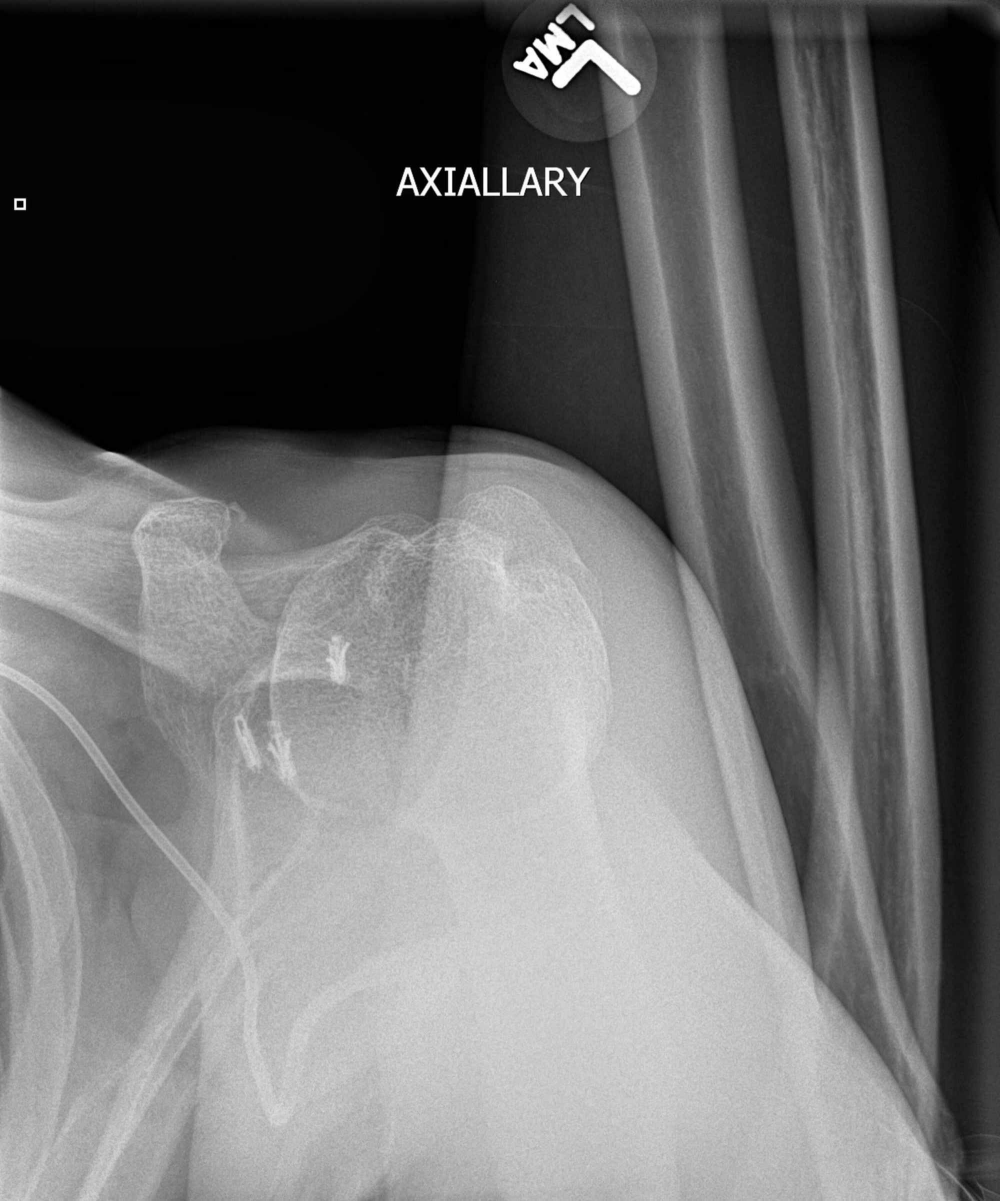
Limited axillary view radiograph of the shoulder showing posteroinferior subluxation of the humeral head

Suture anchors were also seen in the anterior glenoid and the anterior humeral head. A standard AP shoulder radiograph has low sensitivity (50%) for detecting posterior dislocations [1]. The axillary view (Figure 3) is the most reliable method to detect anterior or posterior humeral head subluxation or dislocation.

**Figure 4:**
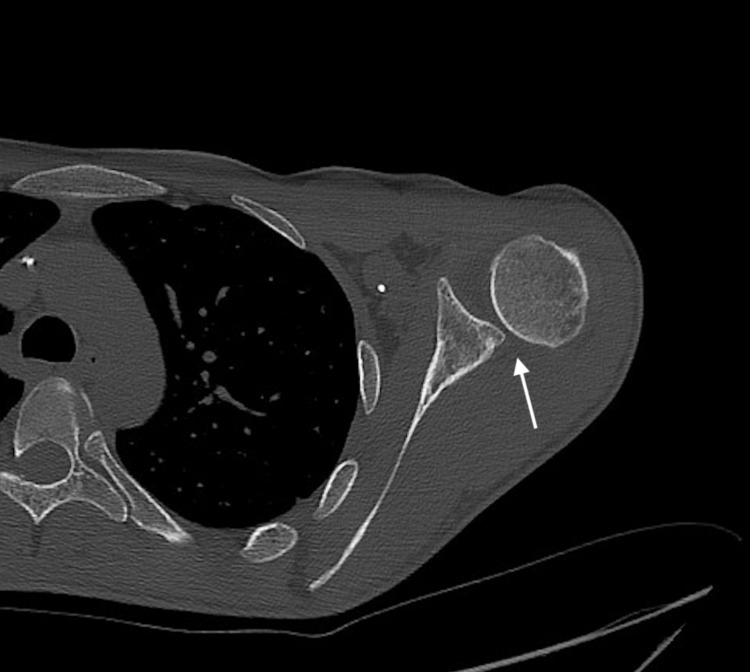
Axial CT shoulder showing posteroinferior subluxation CT: computed tomography

Of note, in the setting of acute dislocation, the patient may not be able to fully abduct the affected upper extremity 90°, limiting the exam. If there is concern over the integrity of the humeral head, glenoid, or ligaments, computed tomography (CT) or magnetic resonance (MR) of the shoulder is indicated. In this case, CT of the shoulder confirms the posteroinferior subluxation of the humeral head relative to the glenoid (Figure 4).

**Figure 5:**
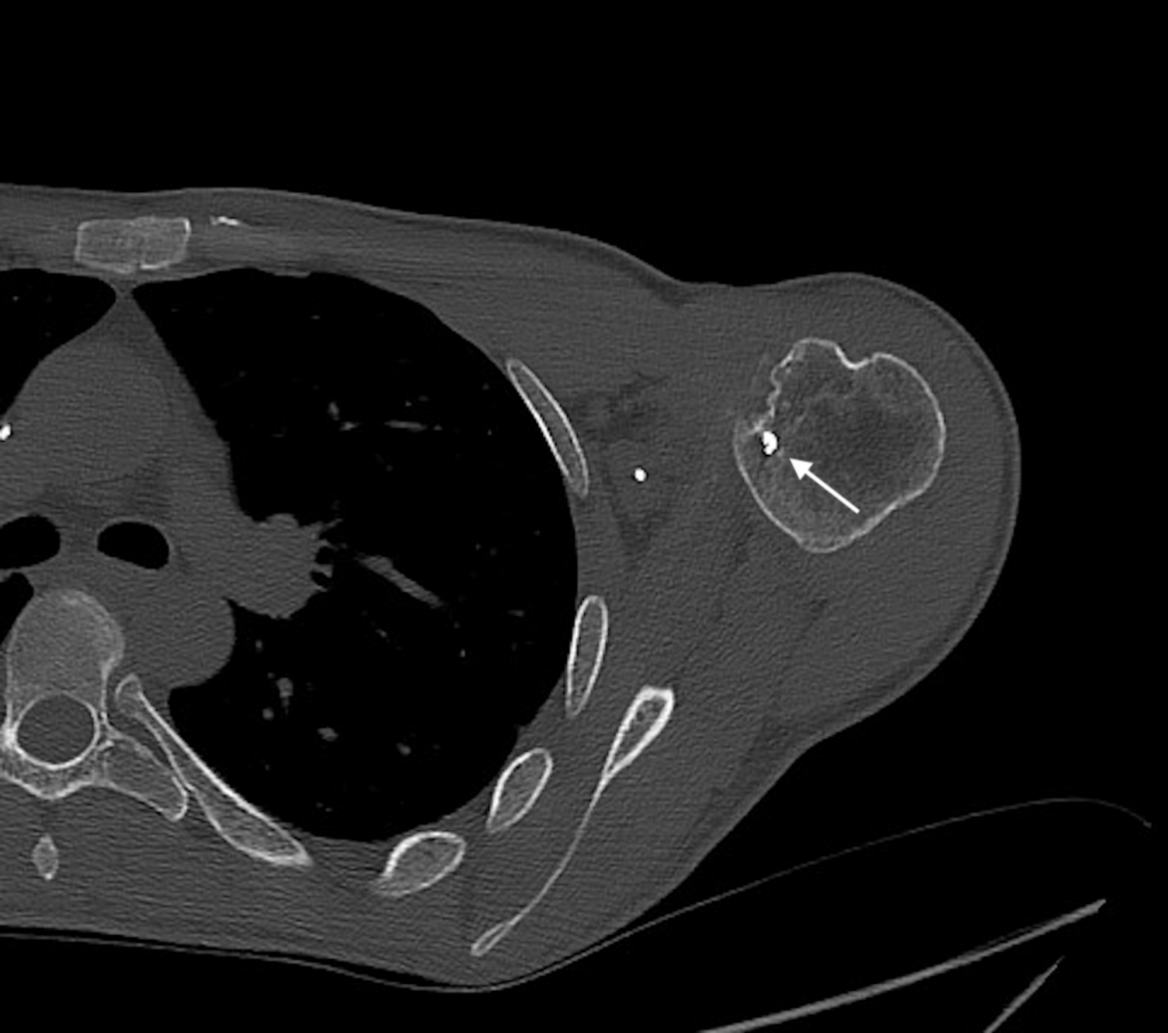
Axial CT shoulder showing an anterior humeral head suture anchor with partially visualized reverse Hill-Sachs deformity CT: computed tomography

**Figure 6:**
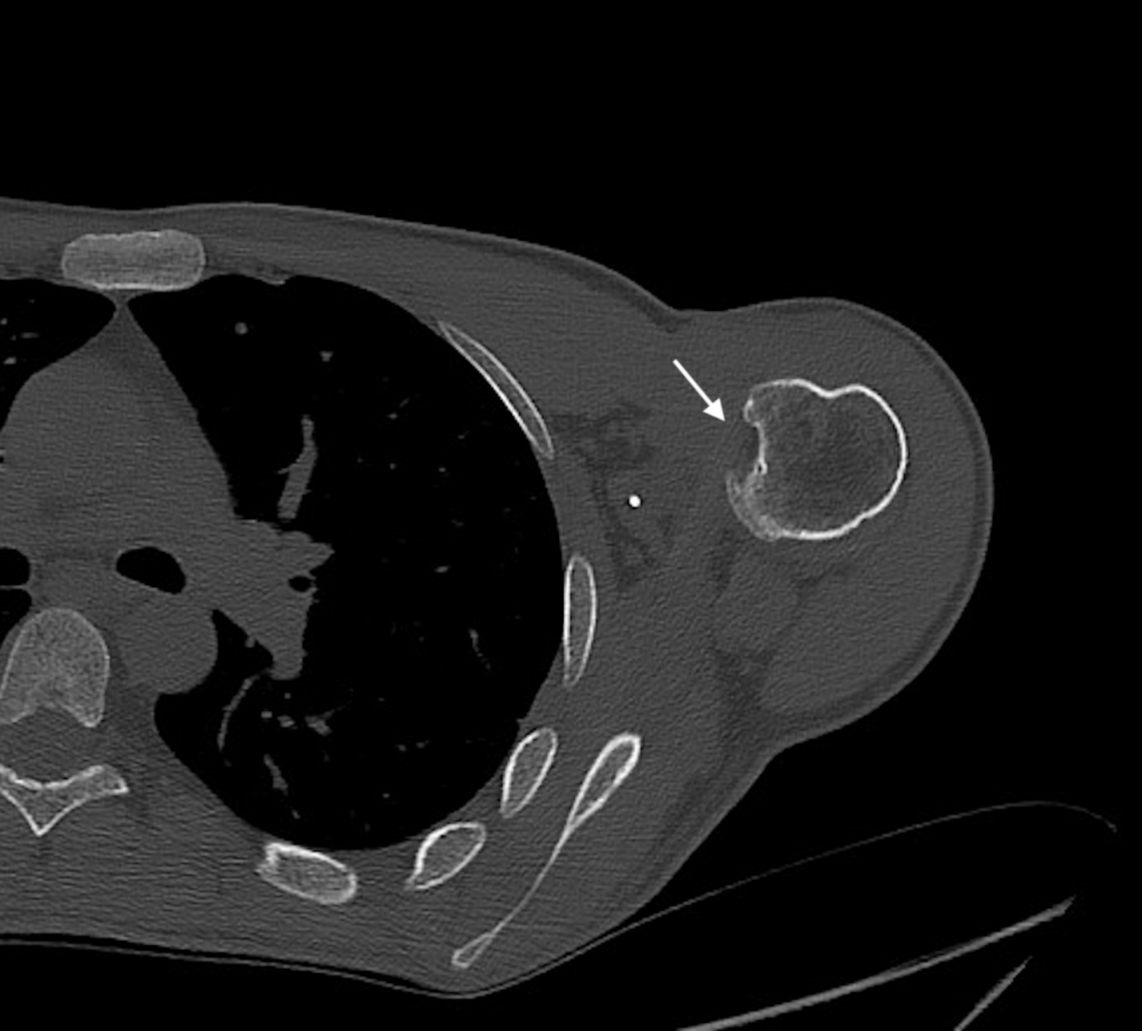
Axial CT shoulder showing a large reverse Hill-Sachs deformity CT: computed tomography

The aforementioned suture anchors are visualized on the CT, as well as on the radiographs (Figure 5). In addition, a 14 mm wide x 7 mm deep vertical defect in the anteromedial aspect of the humeral head was visualized (Figure 6).

**Figure 7:**
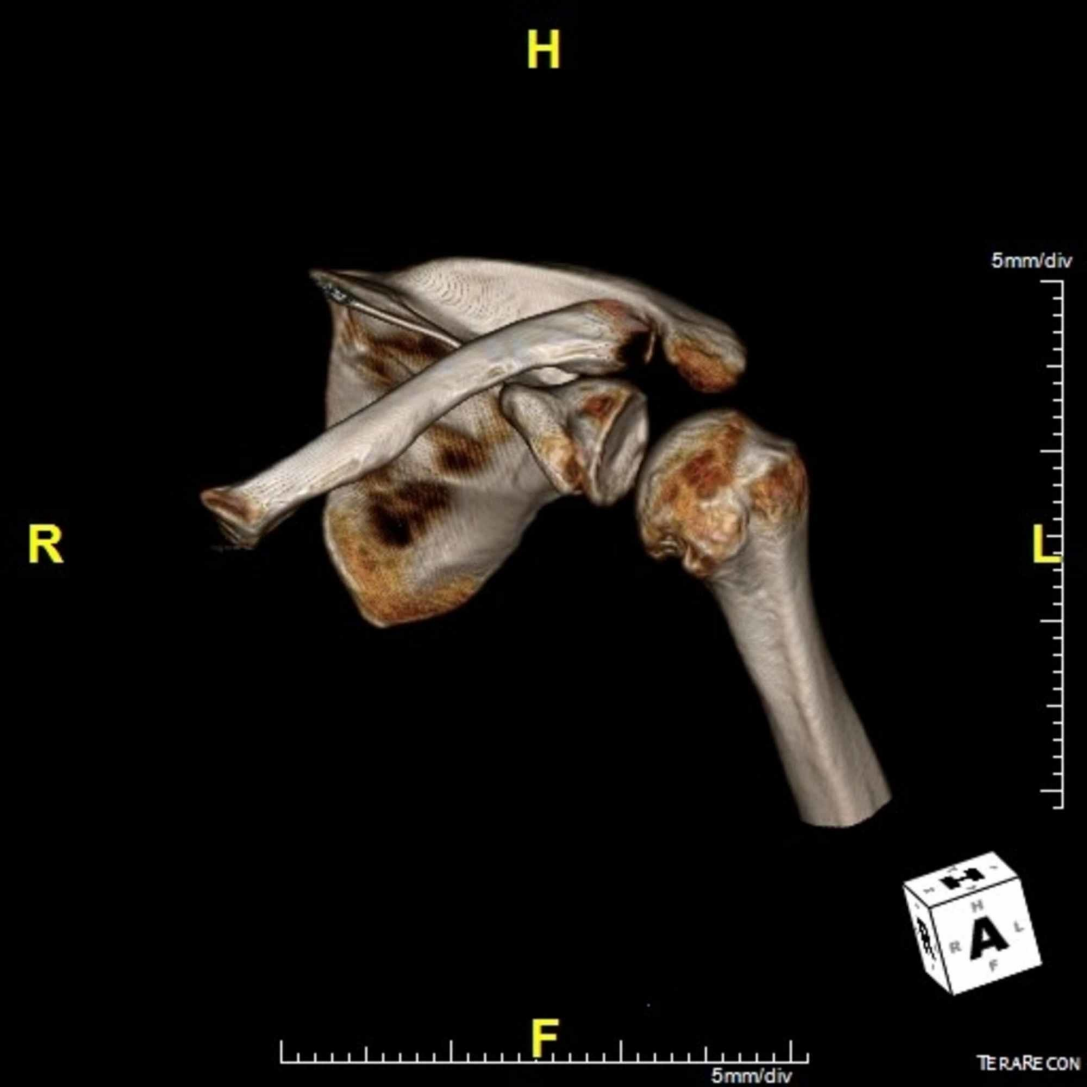
CT 3D reconstruction of the left shoulder again showing a large reverse Hill-Sachs deformity CT: computed tomography; 3D: three-dimensional

In the clinical context of chronic posterior dislocations due to posterior glenohumeral instability, the defect is most likely a reverse-Hill Sachs lesion [7]. This most often occurs secondary to repetitive trauma of the humeral head against the posterior glenoid rim such as during posterior dislocations [7]. Delayed repair of the lesion can lead to avascular necrosis of the humeral head [3]. Although osseous reverse Bankart lesions can coincide with reverse Hill-Sachs lesions, this patient has a well-preserved posterior glenoid rim demonstrated on shoulder CT (Figure 4 and Figure 7) [8].

## Discussion

Posterior shoulder dislocation is an uncommon injury with a prevalence of 1.1 per 100,000 population per year [4]. It generally follows intense contraction of the external rotator muscles and can be secondary to seizure activity, high-velocity trauma, or following an electrical shock. Closed reduction can be utilized following an initial occurrence. Successful reduction is achieved in 96% of cases by longitudinal traction or in the event of a suspected anteromedial humeral head impaction fracture, known as a reverse Hill-Sachs lesion, by manipulation with the arm flexed 90 degrees, internally rotated and adducted [4].

Nonoperative treatment, including physical therapy and rehabilitation, is first-line management for posterior instability with defects involving less than 20% of the humeral head articular surface [2,8]. Surgical treatment is recommended for defects involving 20% to 45% of the humeral head articular surface. Operative management options for posterior fracture-dislocations include open reduction and internal fixation, the McLaughlin procedure, the modified McLaughlin procedure, and hemiarthroplasty [9-10]. Hemiarthroplasty is typically reserved for large articular surface defects, greater than 50%, or other severe damage to the humeral head [11]. The most commonly used method to correct posterior glenohumeral instability with a reverse Hill-Sachs lesion is the modified McLaughlin procedure or McLaughlin type remplissage. The procedure involves dissecting the lesser tuberosity of the humeral head, along with the insertion of the subscapularis tendon, and attaching them to the humeral head defect. This holds the humeral head in constant internal rotation to counteract the immense external rotatory force during seizure activity [8-11]. Currently, these procedures are primarily done arthroscopically [8]. Alternative arthroscopic techniques have now been suggested in addition to the above technique, which include filling the defect with the middle glenohumeral ligament or iliac bone-block autograft [8,12].

Based on the suture anchors located in the anterior glenoid and anterior humeral head (Figures 1-5), the post-surgical changes in our patient are the most consistent with a McLaughlin-type remplissage procedure. Unfortunately for the patient, there was continued laxity following the procedure, allowing for recurrent subluxation.

## Conclusions

This case demonstrates imaging findings associated with the modified McLaughlin type or reverse remplissage procedure for recurrent posterior shoulder instability. Long-term success rates of the procedure are inconclusive due to small sample sizes in the literature. Perhaps a meta-analysis of these studies can shine some light on the overall effectiveness of the procedure.
